# A rare case of thumb polydactyly with metacarpophalangeal joint synostosis

**DOI:** 10.1080/23320885.2019.1596032

**Published:** 2019-04-15

**Authors:** Michael Finsterwald, Sebastian Guenkel

**Affiliations:** Department of Orthopedics and Traumatology, Buergerspital Solothurn, Solothurn, Switzerland

**Keywords:** Thumb duplication, thumb polydactyly, preaxial polydactyly, symphalangism, synostosis, Wassel Type IV, congenital thumb deformity

## Abstract

We report on a rare case of thumb polydactyly with metacarpophalangeal joint synostosis in a 14-year old otherwise healthy boy. Our case can only be classified in the Rotterdam classification, was treated with resection of the hypoplastic radial component and yielded a very satisfactory outcome with a stable thumb.

## Introduction

**Figure 1.**
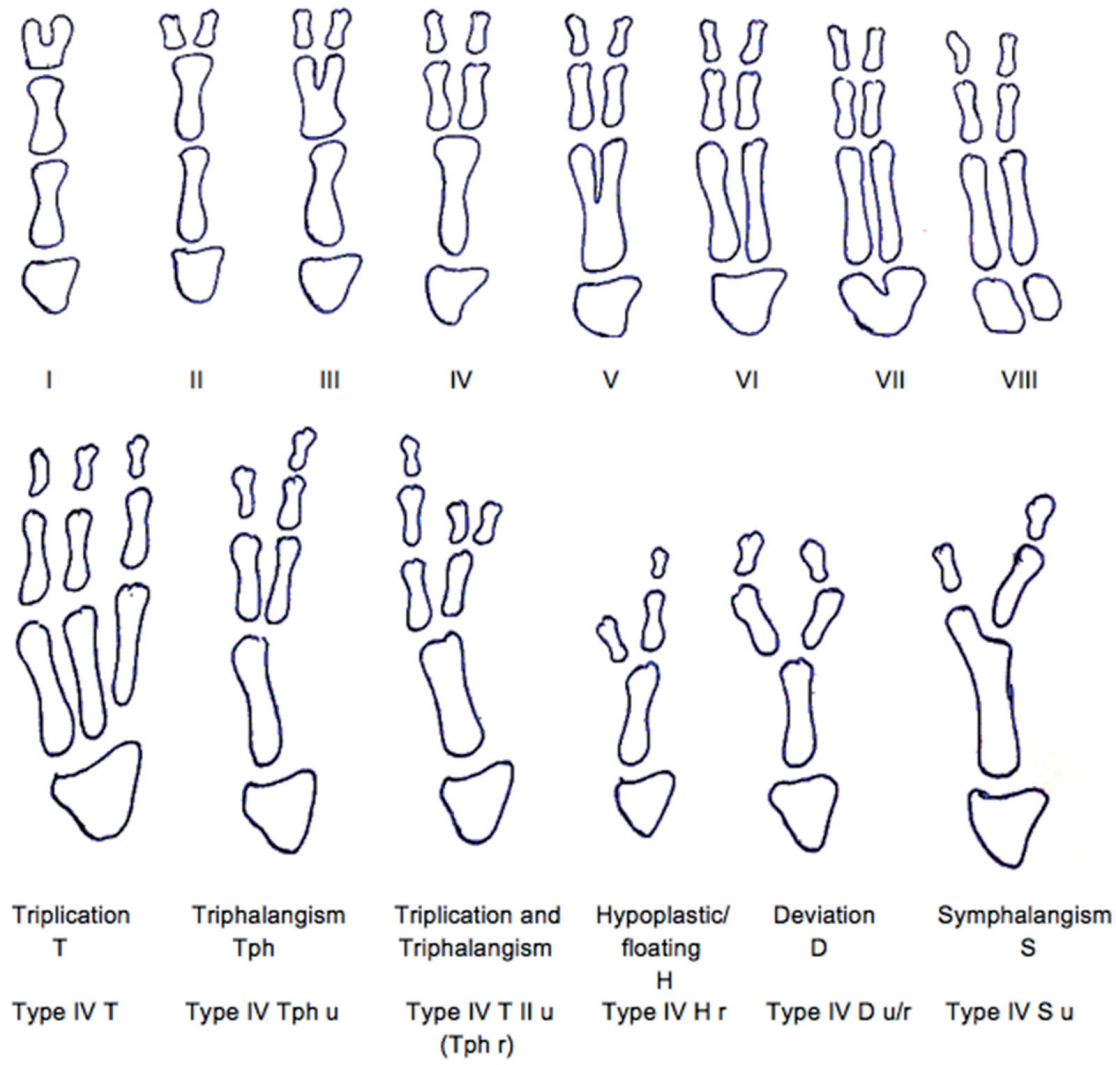
Rotterdam classification (redrawn).

Congenital deformities of the hand affect at least 2.3 in 1000 births [1] with thumb polydactyly being the most common deformity in caucasian and asian people ranging from 0.8 to 1.4 in 1000 births [2,3]. Most of the cases are sporadic and unilateral. There are various classification systems for these deformities. The Wassel system developed in 1969 [4] is still the most widely used. However it is unable to classify some rare deformities and therefore some authors recommend to use the Rotterdam classification [5] (Figure 1), which combines elements of the Wassel [4], the Buck-Gramcko [6] and the Upton [7] taxonomies. According to Dijkman et al [8] 85% of thumb polydactylys are treated with resection and reconstruction, 5% with ablation alone, 8% with the Bilhaut-Cloquet procedure and 1% with pollicization and on-top plasty, respectively.

 

 

## Case report

**Figure 2.**
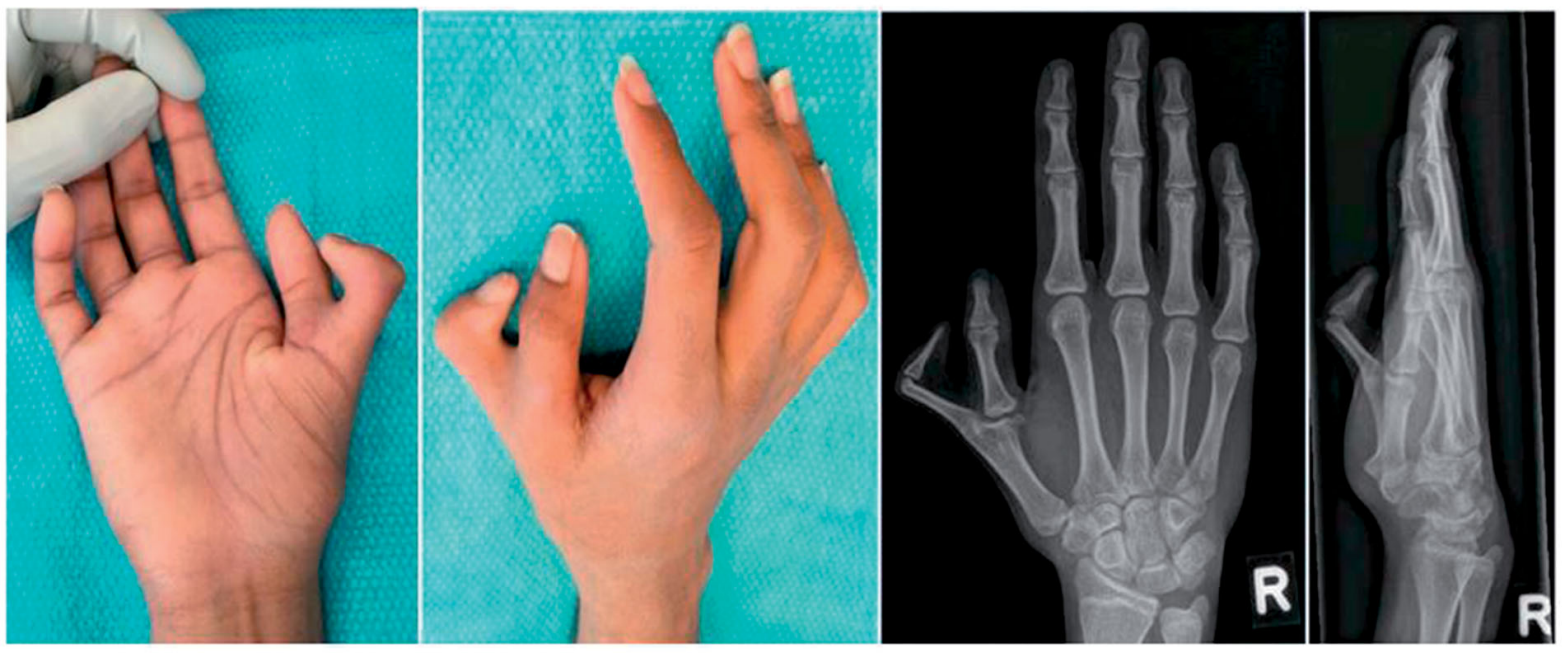
Thumb polydactyly Type IV S u (Rotterdam classification) with radial metacarphophalangeal joint synostosis.

We report on a rare case of thumb polydactyly with metacarpophalangeal joint synostosis which can only be classified with the Rotterdam classification [5]. A 14-year old healthy boy of Ethiopian descent presented with a duplicated thumb on his right hand consisting of a non-functioning hypoplastic radial and a fully developed functional ulnar component (Figure 2). There was no known history of trauma. His left hand appeared to be normal in spite of a small scar radially at the level of the metacarpophalangeal joint, where an extra thumb was presumably strangulated in early childhood. X-Rays of his right hand showed a Wassel Type IV resembling polydactyly with radial synostosis at the metacarpophalangeal joint and a slightly hypoplastic distal phalanx as well as a normally developed ulnar component with a proximal and distal phalanx (Figure 2). Clinical examination revealed normal sensation in both components of the thumb. Assessment of metacarpophalangeal joint stability was not conclusive due to radial synostosis. We therefore planned resection and reconstruction surgery.

**Figure 3.**
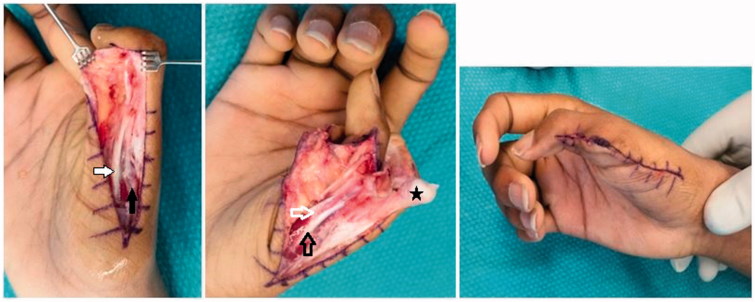
Intraoperative findings, bold white arrow: accessory FPL; bold black arrow: accessory APL; empty white arrow: FPL; empty black arrow: APL; black star: accessory proximal phalanx.

**Figure 4.**
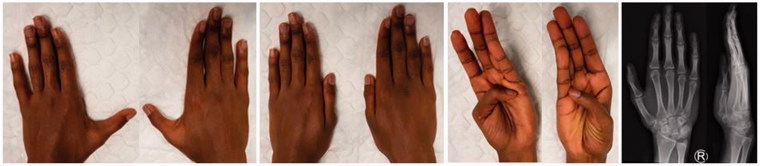
Clinical and radiological result at 1 year postoperative.

During surgery (Figure 3) the hypoplastic bony radial component was resected with an oscillating saw cutting from distal to proximal at the level of the metacarpophalangeal joint and the metacarpal head reduced to match the proximal ulnar phalanx. Flexor and Extensor pollicis longus and brevis tendons of the radial component were dissected proximal of the metacarpophalangeal joint as far as the incision allowed. Careful subperiostal dissection proximally and preservation of the radial collateral ligament and metacarpophalangeal joint capsule distally allowed an anatomic transosseus reinsertion through drill holes with non-absorbable sutures into the distal first metacarpal to stabilise the metacarpal joint against radial stress. In order to avoid a painful neuroma the remaining nerves of the removed radial component were dissected, crushed and buried into an intraosseus drillhole. Clinical examination showed a stable metacarpophalangeal joint with a good range of motion. Postoperatively the thumb was immobilised in a cast for eight weeks. At three months and one year follow-up the patient showed no metacarpophalangeal joint instability, a satisfying motion of the thumb (Figure 4) as well as intact sensation. He was pain free and very pleased with the result. Postoperative and follow-up X-Rays revealed a normal joint alignment.

## Discussion

In the world literature we only found 14 documented cases of thumb malformations with metacarpophalangeal synostosis or interphalangeal symphalangism (Table 1) predominantly from Asia, specifically Saudi Arabia [9,10], Iran [11], Turkey [12] and Japan [13] indicating a potential genetic background. Al-Qattan [10] reported five patients (2.2%) in his study of 228 hands of which only one (0.4%) was a metacarpophalangeal synostosis. Of these five patients three were previously published by Al-Aithan et al [9] and all showed interphalangeous symphalangism. One patient with metacarpophalangeal synostosis and two distal phalanges radially was described by Afshar [11]. Six cases of cartilaginous symphalangism in children were published by Takagi et al [13], three of which were metacarpophalangeal synchondrosis. Boutros et al [14] and Ciloglu et al [12] each reported one case of interphalangeal symphalangism.

**Table 1.** Reported cases of thumb malformations with synostosis at metacarpophalangeal joint (MCPJ) or symphalangism at interphalangeal joint (IPJ).

Author	Type	Region	Comments
Afshar, 2007 [11]	1 MCPJ	Iran	
Al-Qattan, 2010 [10]	1 MCPJ, 4 IPJ	Saudi Arabia	Includes 3 IPJ of Al-Aithan et al, 2005 [9]
Takagi et al, 2009 [13]	3 MCPJ, 3 IPJ	Japan	Cartilaginous in children
Ciloglu et al, 2014 [12]	1 IPJ	Turkey	
Boutros et al, 1998 [14]	1 IPJ	USA	

Even though thumb polydactyly is the most common form of polydactyly in the hand, these cases cannot be classified in the widely used classification by Wassel [4]. Of the multiple modifications of Wassels description, the Rotterdam classification by Zuidam et al [5] is the most complete including bony separation into the carpal bones, thumb triplication as well as synostosis and symphalangism. In a study by Dijkman et al [15], of 520 thumb polydactyly patients only 60% could be classified by the Wassel classification in comparison to 100% by the Rotterdam classification. According to it, our presented case would be classified as Type IV S (symphalangism) u (ulnar).

The reported case is very rare because it consists of a metacarpophalangeal synostosis and two near-normal distal phalanges. Previously reported cases showed either an additional distal interphalangeal joint [11], a slightly deformed interphalangeal joint [10] or symphalangism in more distal joints of the thumb [9,10,12–14]. Interestingly in all reported cases, metacarpophalangeal synostosis or interphalangeal symphalangism was only found in the radial ray.
